# The Development of a Short Form of the Indonesian Version of the Wechsler Adult Intelligence Scale—Fourth Edition

**DOI:** 10.3390/jintelligence11080154

**Published:** 2023-08-04

**Authors:** Christiany Suwartono, Marc P. H. Hendriks, Lidia L. Hidajat, Magdalena S. Halim, Roy P. C. Kessels

**Affiliations:** 1 Faculty of Psychology, Atma Jaya Catholic University of Indonesia, Jakarta 12930, Indonesia; christiany.suwartono@atmajaya.ac.id (C.S.); lidia.hidajat@atmajaya.ac.id (L.L.H.); magdalena.halim@atmajaya.ac.id (M.S.H.); 2 Donders Institute for Brain, Cognition and Behaviour, Radboud University, 6525 GD Nijmegen, The Netherlands; marc.hendriks2@donders.ru.nl; 3 Academic Centre for Epileptology, Kempenhaeghe, 5591 VE Heeze, The Netherlands; 4 Vincent van Gogh Institute for Psychiatry, 5803 DN Venray, The Netherlands; 5 Department of Medical Psychology and Radboudumc Alzheimer Center, Radboud University Medical Center, 6500 HB Nijmegen, The Netherlands

**Keywords:** classification accuracy, psychological assessment, intelligence testing, reliability, short forms, validity

## Abstract

(1) Background: The Wechsler intelligence scales are very popular in clinical practice and for research purposes. However, they are time consuming to administer. Therefore, researchers and psychologists have explored the possibility of shorter test battery compositions. (2) Methods: In this study, we investigated 13 potential short forms of the Indonesian version of the Wechsler Adult Intelligence Scale—Fourth Edition (WAIS-IV-ID). An existing standardization data set of 1745 Indonesian participants collected for the validation of the WAIS-IV-ID was used to examine the short forms’ validity. These ranged from 2-subtest versions to 7-subtest versions. Regression analyses with goodness-of-fit measures were performed, and regression equations were determined for each short form to estimate the Full Scale IQ (FSIQ) score. Discrepancies between the FSIQ and the estimated FSIQ (FSIQ_Est_) scores were examined and classification accuracies were calculated for each short form (% agreement of intelligence classification between the FSIQ_Est_ and FSIQ). (3) Results: None of the 13 short form FSIQ_Est_ values significantly differed from the FSIQ scores based on the full WAIS-IV-ID, and strong correlations were observed between each of these values. The classification accuracies of the short forms were between 56.8% and 81.0%. The 4-subtest short form of the WAIS-IV-ID consisting of the subtests Matrix Reasoning, Information, Arithmetic, and Coding had the optimal balance between best classification values and a short administration duration. The validity of this short form was demonstrated in a second study in an independent sample (*N* = 20). (4) Conclusions: Based on the results presented here, the WAIS-IV-ID short forms are able to reliably estimate the FSIQ, with a significant shorter administration duration. The WAIS-IV-ID short form consisting of four subtests, Matrix Reasoning, Information, Arithmetic, and Coding, was the best version according to our criteria.

## 1. Introduction

The frequent and widespread use of the comprehensive and time-consuming Wechsler intelligence scales for psychological assessments highlight the need for psychologists to explore the possibility of developing shorter batteries of tests. The aim of generating these short forms is to reduce the time required for their administration while maintaining a valid estimate of the Full Scale IQ (FSIQ). Attempts to develop these short forms have been made since the publication of the first version of Wechsler’s intelligence test, the Wechsler–Bellevue Intelligence Scale (McNemar 1950). Short forms have been developed for all the subsequent versions: the Wechsler Adult Intelligence Scale (WAIS) (Doppelt 1956; Maxwell 1957), the Wechsler Adult Intelligence Scale—Revised (Cyr and Brooker 1984; Kaufman et al. 1991; Silverstein 1982; Ward 1990), the Wechsler Adult Intelligence Scale—Third Edition (Donnell et al. 2007; Jeyakumar et al. 2004; Lange and Iverson 2008; Ringe et al. 2002; Ryan and Ward 1999), and the latest version, the Wechsler Adult Intelligence Scale—Fourth Edition (WAIS-IV) (Denney et al. 2015; Fan et al. 2019; Girard et al. 2015; Meyers et al. 2013; Ryan et al. 2015).

From both research and clinical perspectives, the use of the full psychological measurements is recommended; however, participants may have limited time available and tend to not cooperate well if tests are too long. The duration of the test is therefore critical, especially in non-Western cultures in which participants may not be used to lengthy assessments. If researchers need only an estimation of a participant’s overall intellectual ability, yet have limited testing time, a shorter but equally valid short-form test to estimate the FSIQ may be a valid practical choice.

McNemar (1950) suggested that the sample population should not be too homogeneous; therefore, considering the anticipated use of the FSIQ score for screening purposes in the future, this study involved healthy adult participants. Three possible strategies can be used to develop a valid short-form WAIS assessment. The first strategy is to reduce the number of items in all subtests, while the second and third strategies rely on deleting subtests to develop a representative set of subtests to yield an optimal approximation of FSIQ. The second strategy uses a pro-rated calculation of FSIQ based on a selection of subtests. This is usually performed by computing the scaled scores of the administered subtests, followed by multiplying their sum with the total number of subtests, and dividing it by the number of subtests included in the short form. The third strategy relies on a regression-based equation to select the subtests that should be used to estimate FSIQ. While a more extensive review of the different methods to validate short forms of the Wechsler intelligence tests is beyond the scope of this study, these methods—that each have their pros and cons—are reviewed in detail in Jiménez-Bascuñán et al. (2020) and Olivier et al. (2013) (see also King and King 1982; Levy 1968; Silverstein 1990). Short forms that reduce the number of items rather than the number of subtests are less reliable than those that combine four or five subtests (Silverstein 1990); therefore, in this study, we compared several sets of subtests of the WAIS-IV-ID to estimate the FSIQ using the regression-based approach.

In Table 1, we present an overview of the short forms studied here. Since Wechsler’s intelligence scales were originally considered to have a two-factor structure, that is, a verbal and nonverbal (performance) factor of cognitive ability (based on factor analysis on the then-included subtests, see (Gutkin et al. 1984) for a critical discussion), four two-subtest short forms were included, each of which included a verbal factor and a performance factor (SF1–SF4). These two-subtest short forms were all based on previous studies, except for SF4, which was based on the results of our stepwise regression providing a good fit to predict FSIQ. Furthermore, we compared short forms consisting of four, five, six, or seven WAIS-IV subtests, which may better reflect the current four-factor structure of the WAIS-IV (Wechsler 2008b). This four-factor structure has resulted in four index scores: Verbal Comprehension (VC), Perceptual Reasoning (PR), Working Memory (WM), and Processing Speed (PS). Our previous study showed that this four-factor model is a better fit than a five-factor model (Suwartono 2018). Although it should be stressed that our study aim was to examine short versions of the WAIS-IV-ID that provide the best estimate of the FSIQ rather than estimates of the individual index scores, we selected representative subtests from each factor. The selection was thus based on the factor loadings from the four- and five-factor models from our previous study that provided the most adequate Goodness of Fit indexes (Suwartono 2018) and reflect the structure of the Indonesian-language version of the WAIS-IV (WAIS-IV-ID; Suwartono et al. 2014). This resulted in four-subtest version SF6 and five-subtest version SF13. We also added two further short forms based on our data collection: SF5 was developed using the highest independent correlation coefficients between each subtest and the FSIQ, while SF12 combines SF4 and SF5. In addition, we included several short forms based on previous studies of the WAIS short forms, provided that those subtests were still available in the WAIS-IV (SF7–SF11). While studies on the clinical validity of WAIS short forms are scarce, several of the listed SFs have been validated in clinical samples from different countries, such as myotonic dystrophy type 1 (SF2; Garmendia et al. 2022), schizophrenia (SF11; Bulzacka et al. 2016), mild neurocognitive disorder and dementia (SF6, SF8, SF11; Takeda et al. 2018) and traumatic brain injury (SF5; Reid-Arndt et al. 2011). In total, we compared the psychometric properties of 13 short forms with the WAIS-IV-ID.

## 2. Materials and Methods

### 2.1. Sampling Method

In order to identify the best short form for the WAIS-IV-ID, the Indonesian standardization sample was used. All participants were recruited from the Indonesian population using the standardized protocol described in the WAIS-IV manual (Wechsler 2008b). A quota sampling method was used to represent the population census data from the six largest islands in Indonesia; 57.49% (136.6 million individuals) live on Java, 21.31% on Sumatra, 7.31% on Sulawesi, 5.8% on Kalimantan, and 5.50% live on Nusa Tenggara and Bali (Badan Pusat Statistik Indonesia 2012). A second study was conducted to assess the validity of the chosen short form. This validation study was conducted in Jakarta using a convenience sampling method.

### 2.2. Participants

The existing standardization data set of 1745 participants that was collected for the development and construction of the WAIS-IV-ID was used (see Suwartono et al. (2014) for a more detailed description of the data collection). In short, this data set consists of 736 men (42.2%) and 1009 women (57.8%), whose ages ranged from 16.0 to 69.9 years old (*M* = 31.74, *SD* = 14.12). Their education levels were as follows: 8.0% completed only junior high school, 48.6% completed senior high school, 37.0% had obtained an undergraduate degree (BA or BSc), and 6.4% had completed a postgraduate degree (MA, MSc, or PhD). Most of the participants were from Java (58.2%), and the rest were recruited from Sumatra (16.6%), Sulawesi (10.5%), Borneo (8.0%), and Nusa Tenggara and Bali (6.7%).

An independent sample of 20 new participants was recruited for the validation study. The participants were recruited from Atma Jaya University. This convenience sample included 3 men (15%) and 17 women (85%). Their ages ranged from 17 to 60 years old (*M* = 29.80, *SD* = 13.80). Thirteen of these participants completed an undergraduate degree (BA, BSc), 5 completed senior high school, 1 completed junior high school, and 1 completed a Master’s degree. All participants were from Jakarta.

### 2.3. Instruments

The WAIS-IV-ID consists of ten core subtests, Block Design (BD), Similarity (SI), Digit Span (DS), Matrix Reasoning (MR), Vocabulary (VC), Arithmetic (AR), Symbol Search (SS), Visual Puzzle (VP), Information (IN), Coding (CD), as well as five supplemental subtests: Letter–Number Sequencing (LN), Figure Weights (FW), Comprehension (CO), Cancellation (CA), and Picture Completion (PC). The items of the WAIS-IV-ID subtests are the same as or equivalent to those of the US version of the WAIS-IV (Suwartono et al. 2014; Wechsler 2008a). The WAIS-IV-ID has been shown to have a good reliability (Suwartono et al. 2014), structural validity (Suwartono 2018), and external validity (Suwartono et al. 2016). The WAIS-IV-ID is used in Indonesia in the field of education, human resource management, and health care (Suwartono 2018) and replaced the Indonesian adaption of the Wechsler–Bellevue Intelligence Scale, developed in 1939, which was still taught and used in Indonesia until recently (Suwartono et al. 2014).

### 2.4. Procedure

The WAIS-IV-ID was administered individually following the guidelines in the test manual (Wechsler 2008a). All examiners (*N* = 98) were undergraduate Psychology students in the last year of their study or recent Psychology graduates. They were all extensively trained and had passed the test administration course for the WAIS-IV-ID. All participants gave their written informed consent before participating in the study. The research proposal and informed consent forms were approved by all institutions who agreed to participate.

After the best short form was selected, a second study was performed. The new participants were recruited to participate in two test sessions. In the first session, they took the short-form test, while in the second, they took the full WAIS-IV-ID. The interval between the first and second sessions ranged from 27 to 50 days (*M* = 36.7, *SD* = 7.06).

### 2.5. Statistical Analyses

The procedure for determining the subtests used in the short forms was based either on those used in previous research, those indicated by a stepwise regression, or those indicated by independent correlations of subtests with the FSIQ. A regression analysis was performed after selecting the short forms. The goodness of fit for the prediction model was determined using a modified version of *R*^2^ adjusted for the number of predictors in the model (Field 2013). Next, a regression equation was formulated for transforming the standardized subtest scores into an estimated FSIQ score (FSIQ_Est_). The mean discrepancies between the FSIQ and FSIQ_Est_ scores were tested using a dependent *t*-test. The classification accuracy was calculated, which is the percentage agreement of intelligence classification between the FSIQ_Est_ estimated by the short form and the FSIQ determined using the full WAIS-IV-ID (Jones 1967; Levy 1968; Mumpower 1964; Silverstein 1990).

The reliability of each short form was calculated using the composite reliability formula (Crawford et al. 2008; Nunnally and Bernstein 1994; Equation (1)). Here *r_YY_*, is the reliability coefficient of the subtest combination, *k* is the number of component subtests, *r_xx_* is the reliability coefficient of the short form’s components, and *R_y_* is the sum of coefficient correlations in the component correlation matrix.

(1) $$r_{YY}\;=\;1-\;\frac{k\;–\;\Sigma r_{xx}}{Ry}$$

Equation (2) was used to calculate the standard error of measurement (Crawford et al. 2008; Ley 1972). Here, *S_x_* is the standard deviation of the short form and *r_xx_* is its reliability coefficient. The validation of the short forms was determined by the correlation between their FSIQ_Est_ and the FSIQ (Silverstein 1990).

(2) $$\mathrm{SEM}x=S_{x}\sqrt{1-r_{xx}}$$

The unidimensionality of nine models (SF5–SF13) was also tested to examine to what extent they measure general intelligence (*g*). The ω coefficient of reliability considers the factor loadings from a confirmatory factor analysis (CFA) and remains unbiased for uncorrelated errors (Padilla and Divers 2016). The ω reliabilities of the short forms were calculated in *R* following the method outlined by Peters (2014). The ω reliability is based on a hierarchical factor model and can be used with multidimensional scales. A single-factor CFA was performed using the standardized subtest scores with LISREL 8.80 (Jöreskog and Sörbom 2006). The chi-square ratio (χ^2^/df), *p*-value, Root Mean Square Error of Approximation (RMSEA), Akaike’s Information Criterion (AIC), and delta AIC were applied to assess the goodness of fit of the theoretical models with the sample data. Details of these fit indexes can be found in various sources (see Burnham and Anderson 2004; Hu and Bentler 1999; Kline 2005). A good model would provide nonsignificant goodness-of-fit results at a 0.05 threshold (Barrett 2007). RMSEA determines the deviation from a perfect fit; Hu and Bentler (1999) suggested that RMSEA values less than or equal to 0.06 indicate a good fit. The AIC compares different models; smaller AIC values indicate a better fit after accounting for model complexity (Akaike 1987).

The following criteria were applied for the evaluation of the short forms (Levy 1968; Silverstein 1990): the magnitude of correlation between FSIQ and FSIQ_Est_, the mean differences between FSIQ and FSIQ_Est_ revealed by paired sample *t*-tests, and the accuracy of the classification agreement between FSIQ and FSIQ_Est_. The basic requirement for any short form is a minimum correlation of 0.90 with the score of the full assessment (Groth-Marnat 2009).

In the second study, Wilcoxon Signed Rank Tests were used to determine whether the FSIQ and FSIQ_Est_ were significantly different. Spearman’s rho describes the FSIQ_Est_ and FSIQ correlation, in which each subtest in the short forms was represented. To ascertain the reliability of each short form, a composited reliability formula was also used. The standard error of each measurement was calculated.

## 3. Results

Table 2 shows the results of all the criteria used to evaluate the short forms. All of the regression analyses on the various short forms indicated that they significantly predicted FSIQ. The goodness of fit of our prediction models in estimating the FSIQ was calculated with an adjusted *R*^2^, which ranged from 0.60 (SF3) to 0.94 (SF11). The short forms that adequately predict FSIQ should explain more than 90% of the variance in FSIQ_Est_. Based on the adjusted *R*^2^, we found that SF5, SF11, SF12, and SF13 accurately predicted FSIQ. For all of the short forms, the correlations between FSIQ and FSIQ_Est_ were significant (*p* < .01), ranging from 0.77 (SF3) to 0.97 (SF11). However, only SF5–SF13 had correlation coefficients higher than 0.90, with an explained variance over 81%. Next, we tested whether the FSIQ and FSIQ_Est_ values (based on the regression models) were significantly different using a paired *t*-test analysis. None of the FSIQ_Est_ scores from the short forms were significantly different from the FSIQ value.

The third evaluation was a determination of classification accuracy. Classification accuracy is the number of correct predictions made, divided by the total number of predictions made, multiplied by 100 to turn it into a percentage. We classified the FSIQ and FSIQ_Est_ for each participant using a manual, then compared whether they were classified into the same IQ score category. The classification accuracy of the FSIQ_Est_ values of each short form compared with the FSIQ ranged from 56.79% (SF3) to 81.03% (SF11). We found that SF5, SF11, SF12, and SF13 were the four short forms with the highest classification accuracies.

Table 3 shows the reliability parameters for all short forms. The composite reliability coefficients ranged from 0.82 (SF2) to 0.95 (SF12 and SF13). The short forms comprised of four or more subtests (SF5–SF13) had composite reliability coefficients above 0.90, although this was also achieved by SF4, which consists of two subtests (MR and CD) and had a coefficient of 0.91. The ω reliability coefficients of the short forms with four or more subtests ranged from 0.69 (SF10) to 0.81 (SF12).

SF5, SF7, and SF8 had insignificant Chi-square test values (*p* > .01) for their structural validities. Table 4 shows the factor loadings and average variances extracted for these three well-fitting short forms. These results indicate that the subtests included in these short forms were sufficiently able to measure IQ as a unidimensional construct. Moreover, these short forms fulfilled the criteria for RMSEA (RMSEA < 0.06), for which smaller values indicate a better model fit and predicted values close to the observed data values.

Considering the evaluation criteria from Table 2, we concluded that SF5 is the best short form test for the WAIS-IV-ID. SF5 produced the following values: χ^2^/df = 2.91, *p* > .01, RMSEA = 0.03, AIC model = 21.82, and delta AIC = 2.60. This choice was further supported by combining the results of the evaluation criterion, test reliability, and structural validity. In SF5, IN represents the Verbal Comprehension, MR the Perceptual Reasoning factor, AR the Working Memory factor, and CD the Processing Speed factor. The FSIQ_Est_ for SF5 can be computed based on the scaled scores for the four subtests using Equation (3) (cf. Meyers et al. 2013).

FSIQ_Est_ = 46.20 + 1.54 × IN + 1.37 × MR + 1.08 × AR + 1.32 × CD (3)

A second study was performed to assess the validity of using SF5 as a short form of the WAIS-IV-ID. A Wilcoxon Signed Rank test indicated no significant difference between the FSIQ calculated by the full test and the FSIQ_Est_ values predicted using SF5 (*Z* = −1.68, *p* = .09). The time taken to administer the four SF5 subtests was 15–38 min (*M* = 25.45, *SD* = 5.36). The SF5 classification accuracy was 70%, while its composite reliability coefficient was 0.94 (*SEM* = 2.73). Spearman’s rho correlation between FSIQ and the FSIQ_Est_ predicted with SF5 was *r*(18) = 0.89, *p* < .01. Each subtest in the short form correlated significantly with FSIQ, ranging from 0.59 (CD) to 0.76 (AR). The largest correlation coefficients for each index were IN with VCI (*r*(18) = 0.83, *p* < .01), MR with PRI (*r*(18) = 0.71, *p* < .01), AR with WMI (*r*(18) = 0.74, *p* < .01), and CD with PSI (*r*(18) = 0.83, *p* < .01). More details about the correlations between the short-form and Full Scale indexes can be found in Table 5.

## 4. Discussion

In the current study, we examined the reliability and convergent validity of 13 short forms of the WAIS-IV-ID, which consisted of two, four, five, six, or seven subtests. Overall, no significant differences were observed between the FSIQ value determined using the full test and the FSIQ_Est_ values predicted using the short forms. For the short forms comprising two subtests, we found that SF4 (subtests MR and CD) yielded the best estimate of the Full Scale IQ. This result does not support the findings of Denney et al. (2015) using the WAIS-IV-US, who reported that SF2, consisting of VC and BD, was the two-subtest short form with the best fit. In our study, SF4 had a higher classification accuracy, correlation with the FSIQ, and reliability than SF2. Here, it should be noted that classification of IQ scores is based on arbitrary criteria (i.e., strata of 10 IQ points) which nonetheless reflect the consensus in the field (see, e.g., Groth-Marnat 2009; Wechsler 2008b). However, the classification accuracy has no intrinsic meaning and is included for descriptive purposes, as in (clinical) practice, decisions are often based on the verbal classification labels.

All short forms consisting of four subtests yielded reliable FSIQ_Est_ values; however, only SF5, SF7 (McNemar 1950), and SF8 (Kaufman et al. 1991) had satisfactory goodness-of-fit index results (χ^2^/df, *p* > .01, RMSEA < 0.06, AIC model). Our findings suggest that SF5 (subtests IN, MR, AR, and CD) had the highest predictive value (based on the adjusted *R*^2^ value), percentage of classification accuracy, and coefficient of reliability among the four-subtest short forms. For the short forms that consist of five or more subtests (SF10–SF13), the goodness of fit index results were unsurprisingly not satisfactory (χ^2^/df, *p* < .01, RMSEA > 0.06, AIC model), as more added subtests will, by definition, result in a poorer representation of a unidimensional model.

Decisions on which subtests to include may depend on the type of information required (Groth-Marnat 2009). When time limitation is the primary factor for reducing the evaluation, short forms containing more than four subtests may not be an ideal solution (Denney et al. 2015). Furthermore, the subtests MR, IN, AR, and CD that make up SF5 have a short administration duration, include a test from each of the four index scores, are easy to administer, and require little subjectivity in scoring. This was corroborated by our second study, showing that the time required to administer SF5 was approximately 25 min. We, therefore, recommend the use of Reasoning, Information, Arithmetic, and Coding (SF5) in place of the full WAIS-IV-ID to reduce the time required while retaining the maximum validity. Our findings are also in agreement with another recent study using the Taiwanese version of the WAIS-IV (Chen and Hua 2020). Here, the psychometric properties of all 90 possible tetradic short forms were studied using the full battery in a sample of 1105 healthy individuals between 18 and 90 years of age. The FSIQ_Est_ based on the subtests Matrix Reasoning, Information, Arithmetic, and Coding short form was also found to have excellent equivalence with the FSIQ in that study. This version was also among the ten 4-subtest versions with the shortest administration duration (i.e., 24–25 min).

A strength of the current study is the use of a large standardization sample. The development of short forms is usually based on smaller clinical samples, as was the case for the short forms of the original US version of the WAIS-IV (Denney et al. 2015; Girard et al. 2015; Meyers et al. 2013; Ryan et al. 2015). We also included several recently proposed short forms based on our findings in previous research (Suwartono 2018). Of these, SF5 best estimated the FSIQ. Our second study investigated the psychometric properties of SF5, with promising results. However, the sample size of our second study is small and recruitment bias may have occurred, as participants were recruited via Atma Jaya University. Further research is thus needed to replicate these findings in a larger sample population. Furthermore, future studies should investigate the convergent and divergent validity, the known-group validity (e.g., using clinical populations), and cross-validate the findings in, for instance, different age groups.

The current study has other limitations. We developed our short forms based on data obtained from a sample who completed the full WAIS-IV battery of tests. If the motivation and attention of the participants varied during the administration of the full test, these scores may have affected the selection of subtests used to estimate FSIQ_Est_ (Thompson 1987). Further research should investigate the validity of the short forms in an independent sample, and examine whether the classification agreement rates remain high, indicating the best trade-off between a reduced administration time and a potential loss in reliability and validity (Girard et al. 2015; Smith et al. 2000). Furthermore, since the introduction of the WAIS-IV it has been possible to compute the General Ability Index (GAI), which is based on six subtests and relies on the crystallized and fluid ability factors VC and PR. The GAI has been argued to also be a time-efficient estimate of *g,* which, in addition, is also less susceptible to the effects of brain dysfunction (Tulsky et al. 2001). However, the use of the GAI is, to date, not as widely accepted as the FSIQ. Also, the administration duration of the GAI subtests is considerably longer than the four subtests that make up SF5, since BD is a subtest with a long administration duration (Axelrod 2001).

It should be noted that the results obtained using any short form should be interpreted with caution, as these only represent an estimate of FSIQ (King and King 1982; Silverstein 1990). Our study was also only aimed at identifying the short form of the WAIS-IV-ID that best predicted the FSIQ. Consequently, even though the best version (SF5) includes tests from all four factors, it should neither be used to estimate the Index Scores nor for performing profile analyses of individual participants. Moreover, as the results in Table 2 show, differences of five IQ points or more between FSIQ_Est_ and FSIQ are not rare, even for the SFs with the best psychometric characteristics. Short forms are thus best used to obtain a quick indication of intelligence to determine whether an additional (neuro)psychological assessment is required (Groth-Marnat 2009). In addition, the short forms might be useful for research in which individual classifications or absolute FSIQs do not have diagnostic consequences (Kaufman and Kaufman 2001).

## Figures and Tables

**Table 1:** Model specifications for the various short forms of the Wechsler Adult Intelligence Scale—Fourth Edition.

Short Forms	Subtests ^1^	Basis of the Model
SF1	IN and BD	Maxwell (1957); Ringe et al. (2002)
SF2	VC and BD	Cyr and Brooker (1984); Denney et al. (2015); Maxwell (1957); Ringe et al. (2002); Silverstein (1982)
SF3	IN and PC	Kaufman et al. (1991)
SF4	MR and CD	Stepwise regression method
SF5	IN, MR, AR, and CD	Correlations with FSIQ
SF6	IN, MR, DS, and CD	The best-fit four-factor model (Suwartono 2018)
SF7	CO, BD, AR, and CD	McNemar (1950)
SF8	SI, PC, AR, and CD	Kaufman et al. (1991)
SF9	DS, AR, SS, and CD	Wechsler (2008b; CPI)
SF10	SI, VC, IN, BD, MR, and VP	Wechsler (2008b; GAI)
SF11	SI, IN, BD, PC, DS, AR, and CD	Ward (1990)
SF12	IN, MR, DS, AR, and CD	Combination of SF4 and SF5
SF13	IN, MR, VP, DS, and CD	The best-fit five-factor model (Suwartono 2018)

^1^ IN = Information; BD = Block Design; VC = Vocabulary; PC = Picture Completion; MR = Matrix Reasoning; CD = Coding; AR = Arithmetic; DS = Digit Span; CO = Comprehension; SI = Similarity; SS = Symbol Search; VP = Visual Puzzle.

**Table 2:** Results on the criteria to evaluate each short form.

Short Forms	Adjusted *R*^2^	Correlation between FSIQ and FSIQ_Est_ (*r*-Value)	Difference between FSIQ and FSIQ_Est_ (*t* Value)	Significance of Difference (*p* Value)	Classification Accuracy (%)	Median Absolute Difference between FSIQ and FSIQ_Est_	Mean Absolute Difference between FSIQ and FSIQ_Est_	SD Absolute Difference between FSIQ and FSIQ_Est_	Min Absolute Difference between FSIQ and FSIQ_Est_	Max Absolute Difference between FSIQ and FSIQ_Est_	Percentage of Differences Surpassing 5 Points	Percentage of Differences Surpassing 10 Points
SF1	0.65	0.81 **	1.59	0.11	58.45	5	6	4.60	0	30	48.40	17.39
SF2	0.66	0.81 **	0.31	0.76	59.26	6	7	4.98	0	26	50.92	20.31
SF3	0.6	0.77 **	0.03	0.97	56.79	4	5	3.73	0	26	36.44	8.87
SF4	0.78	0.88 **	0.03	0.97	67.62	3	3	2.51	0	13	17.68	1.03
SF5	0.91	0.95 **	−0.25	0.81	77.08	3	3	2.59	0	14	19.22	1.77
SF6	0.9	0.95 **	0.28	0.78	75.76	3	4	2.93	0	18	24.60	3.03
SF7	0.87	0.93 **	0.13	0.9	71.75	3	4	3.18	0	20	25.86	4.18
SF8	0.85	0.92 **	−0.59	0.55	72.49	2	3	1.98	0	12	8.92	0.46
SF9	0.83	0.91 **	−0.5	0.62	69.4	3	4	2.87	0	21	22.60	2.86
SF10	0.87	0.93 **	−0.95	0.34	75.3	4	4	3.34	0	19	30.43	6.06
SF11	0.94	0.97 **	0.97	0.33	81.03	3	3	2.24	0	13	13.04	0.57
SF12	0.92	0.96 **	−0.07	0.94	79.08	5	6	4.56	0	26	48.28	17.51
SF13	0.93	0.96 **	−0.74	0.46	79.48	2	3	2.14	0	13	10.81	0.46

** Correlation is significant at the 0.01 level (two-tailed).

**Table 3:** Reliability and validity of the individual models.

Short Form	Reliability		SEM ^3^	Goodness-of-Fit Model				
	Composite ^1^	ω ^2^		χ^2^/df	*p*	RMSEA	AIC Model	Delta AIC
SF1	0.85	not applicable	4.02	not applicable				
SF2	0.82	not applicable	4.40	not applicable				
SF3	0.88	not applicable	3.52	not applicable				
SF4	0.91	not applicable	3.37	not applicable				
SF5	0.94	0.77	3.03	2.91	.05	0.03	21.82	2.60
SF6	0.94	0.72	3.08	20.51	.00	0.11	57.01	37.79
SF7	0.91	0.71	3.69	3.54	.03	0.04	23.07	3.85
SF8	0.91	0.72	3.68	1.61	.20	0.02	19.22	0
SF9	0.92	0.73	3.25	111.39	.00	0.25	238.78	219.56
SF10	0.94	0.69	3.05	60.16	.00	0.18	565.43	546.21
SF11	0.94	0.72	3.02	20.70	.00	0.11	317.74	298.52
SF12	0.95	0.81	2.83	11.82	.00	0.08	79.11	59.89
SF13	0.95	0.73	2.90	25.18	.00	0.12	145.92	126.7

^1^ Based on the composite reliability (Crawford et al. 2008; Nunnally and Bernstein 1994); ^2^ based on the Omega hierarchical value (Peters 2014); ^3^ based on the composite reliability coefficient.

**Table 4:** Factor loadings (λ) and average variance extracted (AVE) for the three well-fitting SFs based on the Chi-square test.

Short Form	Subtest	λ	AVE
SF5	IN	0.67	0.50
	MR	0.77	
	AR	0.78	
	CD	0.58	
SF7	CO	0.55	0.42
	BD	0.60	
	AR	0.77	
	CD	0.60	
SF8	SI	0.61	0.42
	PC	0.61	
	AR	0.69	
	CD	0.64	

**Table 5:** Correlation between the short form and Full Scale IQ of WAIS-IV-ID.

SF5	FSIQ	VCI	PRI	WMI	PSI
FSIQ_Est_	0.89 **	0.74 **	0.68 **	0.78 **	0.58 **
IN	0.69 **	0.83 **	0.41	0.60 **	0.49 *
MR	0.67 **	0.49 *	0.71 **	0.49 *	0.29
AR	0.76 **	0.58 **	0.61 **	0.74 **	0.20
CD	0.59 **	0.50 *	0.31	0.47 *	0.83 **

** Correlation is significant at the 0.01 level (two-tailed); * correlation is significant at the 0.05 level (two-tailed).

## Data Availability

Data are available upon reasonable request from the first author.
